# Cross-sectional investigation of mycological diagnosis challenges in Saudi Arabia

**DOI:** 10.3389/fcimb.2023.1203892

**Published:** 2023-06-12

**Authors:** Aiah Mustafa Khateb, Shatha Ali Alkhaibari

**Affiliations:** ^1^ Department of Medical Laboratory Technology, Collage of Applied Medical Science, Taibah University, Medina, Saudi Arabia; ^2^ Special Infectious Agents Unit, King Fahd Medical Research Center, King Abdulaziz University, Jeddah, Saudi Arabia

**Keywords:** Saudi Arabia, diagnosis, mycology, *Candida*, *Aspergillus*, antifungal, susceptibility, resistance

## Abstract

**Background:**

The global incidence of fungal infection has increased dramatically over the last two decades. Fungal diseases threaten both immunocompetent, and immunocompromised patients. The current fungal diagnostics status in Saudi Arabia needs to be evaluated, especially with the increase of the immunosuppressed population. This cross-sectional study investigated the gaps in mycological diagnosis on a national level.

**Materials and methods:**

The call interview questionnaire responses were collected to evaluate the demand for fungal assays, diagnostic methods’ quality, and mycological expertise of laboratory technologists in both public and private medical intuitions. The data were analyzed using (IBM SPSS ^®^ software version 22.0).

**Results:**

A total of 57 hospitals from all Saudi regions participated in the questionnaire; however, only 32% received or processed mycological samples. Most participants were from the Mecca region (25%), Riyadh region (19%), and Eastern region (14%). The top fungal isolates identified were *Candida* spp., *Aspergillus* spp., and dermatophyte. Fungal investigation is highly requested by intensive care, dermatology, and obstetrics and gynecology units. Most laboratories rely on fungal culture and microscopic examination, which mostly identify *Candida* to the genus level, and use 37°C incubators for culture (67%). Antifungal susceptibility testing (AST) and serological and molecular methods are rarely performed and mostly outsourced. Using accurate identification and AST are the primary factors to improve fungal diagnosis in respect to turnaround time and cost. The three major obstacles identified were availability of facility (47%), reagents and kits (32%), and good training (21%).

**Conclusions:**

The results indicated that fungal diagnosis demand was relatively higher in high-population regions. This study highlighted the gaps in fungal diagnostics reference laboratories to encourage their improvement in Saudi hospitals.

## 1 Introduction

Fungal infections are raising across the globe due to the organism’s adaptation (Pfaller and Diekema, 2007; Bongomin et al., 2017). Despite the use of advanced diagnostic assays, fungal diagnosis, and differentiating colonization from infection, fungal diagnosis remains a mystery. A variety of challenges contribute to the clinical diagnosis of fungal pathogens (Wickes and Romanelli, 2020). A Saudi study reported 11 cases of fungal infection by *Basidiobolus ranarum* that were misdiagnosed as cancer, inflammatory bowel diseases, and granulomatous diseases (Alshehri, 2013). The clinical symptoms are marked by their ambiguity and have high similarity with other diseases. Furthermore, early diagnosis and the optimal management of these infections require a high degree of suspicion, reliable diagnostic tests, and a strong educational background supported by extensive training in medical mycology (Pfaller and Diekema, 2007). Increased resistance and virulence are observed in both clinical and environmental strains. A tertiary care center in Saudi Arabia investigated the changing pattern of *Candida* species from 2003 to 2012, reporting that invasive *Candida* infections caused by *Candida albicans (C. albicans)* remained steady throughout time with a significant increase of *Candida glabrata* (*C. glabrata*) (Omrani et al., 2014). In 2020, Saudi studies reported *C. glabrata* as the most frequent resistant strain to antifungal treatments of vulvovaginal candidiasis (VVC) and candidemia with extended hospital stay (Aldardeer et al., 2020; Yassin et al., 2020). Certain fungal species can cause serious outbreaks; thus, continuous surveillance and identifying the causative agents at the species level and measuring the resistance level are crucial. The *Candida* multidrug-resistant species known as *Candida auris* has caused outbreaks globally and continues to cause outbreaks in Saudi hospitals (Alshamrani et al., 2021). *C. auris* can persist for numerous weeks on patients’ skin and may cause outbreaks, which explains the elevated threat in a hospital’s healthcare settings (Chybowska et al., 2020; Meyer et al., 2021). According to Centers for Disease Control and Prevention (CDC), due to the lack of laboratory equipment and wide identification capability, fungal infections caused by *C. auris* are difficult to confirm, which might lead to misdiagnosis (CDC, 2019). Conventional methods such as microscopic examination and culture require higher experience to interpret the fungal species level. Advanced diagnostic methods increase the capability of fungal detection with minimum experience compared to conventional methods. However, financial obstructions result in the limited availability of these diagnostic instruments.

There is an urgent need to enhance the awareness of healthcare personnel of these emerging fungal pathogens and to identify its prevalence, impact, and resistance, specifically at a national level. To offer accurate diagnosis and execute early control measures to prevent hospital outbreaks, enhanced laboratory testing procedures are required to identify fungal infections (Lu et al., 2018). The current fungal diagnostic capability is unknown in Saudi Arabia as there are no current data on the diagnostic capacity. Saudi Arabia was not included in the large survey of seven Asian countries for fungal diagnostic capabilities (Chindamporn et al., 2018; Wang et al., 2020). In this study, we investigated the demand for mycological diagnostic services in Saudi Arabian medical centers by call interviews designed to evaluate the tests ordered, diagnostic methods’ quality, and the mycological background of the laboratory technologists.

## 2 Material and methods

### 2.1 Study population

This cross-sectional study was targeted at microbiology/mycology laboratory technologists working in the Kingdom of Saudi Arabia (KSA). We used a combination of phone calls, survey invitations, and snowball samplings to recruit the targeted sample. The invitation letter was distributed through official channels and professional networks. After collecting all hospitals numbers from the Ministry of Health (MOH) website, private invitations were sent to private hospitals and laboratories (approximately 450 hospitals in Saudi Arabia). The recommended sample size was 208 or more measurements/surveys in order to attain a confidence level of 95% and a real value within ±5% of the surveyed value. We contacted a total of 280 hospitals (227 government hospitals and 53 private hospitals).

### 2.2 Survey design and data collection

A semi-structured questionnaire consisting of 32 questions was designed and categorized into five sections: demography, sample evaluation, laboratory space and equipment, fungal diagnostic tests, and manpower evaluation (**Table S1**). The design of the questionnaire was based on previously published papers that were closely related to our topic (Wang et al., 2020; Driemeyer et al., 2022). The questionnaire was pretested, reviewed, and validated by experts before data collection. The data were collected by a call interview in March 2022, conducted from 10 am to 3 pm on weekdays. The data were analyzed using IBM SPSS ^®^ software version 22.0 to identify and test the normality of all variables. Categorical data were presented as frequencies.

## 3 Results

### 3.1 Population demographics

There was a total response from 57 hospitals. The study included representative hospitals from all 13 Saudi regions. The majority of the participants were from the Mecca region (25% (n= 14)), followed by the Riyadh region (19% (n= 11)), Eastern region (14% (n= 8)), Northern Borders region (9% (n= 5)), Medina region (8% (n= 4)), Jizan region (5% (n= 3)), Hail region (5% (n= 3)), Al-Baha region (4% (n= 2)), Asir region (4% (n= 2)), and Tabuk region (4% (n= 2)), while the Al-Qassim, Najran, and Al-Jouf regions were 1 (2%) each. Most participating hospitals were from the private sector (60% (n= 34)), while 40% (n= 23) were public hospitals, as shown in (**Table 1**). Only 28% (n=15) of these hospitals had a microbiology laboratory that processed mycological samples, and 68% (n= 39) of them did not process mycological samples at all or used external services. Most data presented in the section below are from the 32% (n=18) of hospitals that were able to answer most of the questions regarding fungal detection. Most of these laboratories used external quality control schemes or accreditation, including the Saudi Central Board for Accreditation of Healthcare Institutions (CBAHI) (n=15, 60%), Joint Commission International (JCI) (n=5, 20%), College of American Pathologists (CAP) (n=2, 8%), and few that were not accredited (n=3, 12%) (**Table 1**).

**Table 1:** Demography of participating hospitals.

	Number response (n) percentage (%)
Demography	
Processed mycological samples in-house	
Yes	18 (32%)
No	39 (68%)
KSA Regions	
Mecca region	14 (25%)
Riyadh region	11 (19%)
Eastern region	8 (14%)
Northern region	5 (9%)
Medina region	4 (8%)
Hail region	3 (5%)
Jizan region	3 (5%)
Al-Baha region	2 (3%)
Asir region	2 (3%)
Tabuk region	2 (3%)
Al-Qassim region	1 (2%)
Najran region	1 (2%)
Al-Jouf region	1 (2%)
Institution type	
Private hospital	34 (60%)
Public hospital	23 (40%)

### 3.2 Fungal load and samples assessment

**Figure 1:**
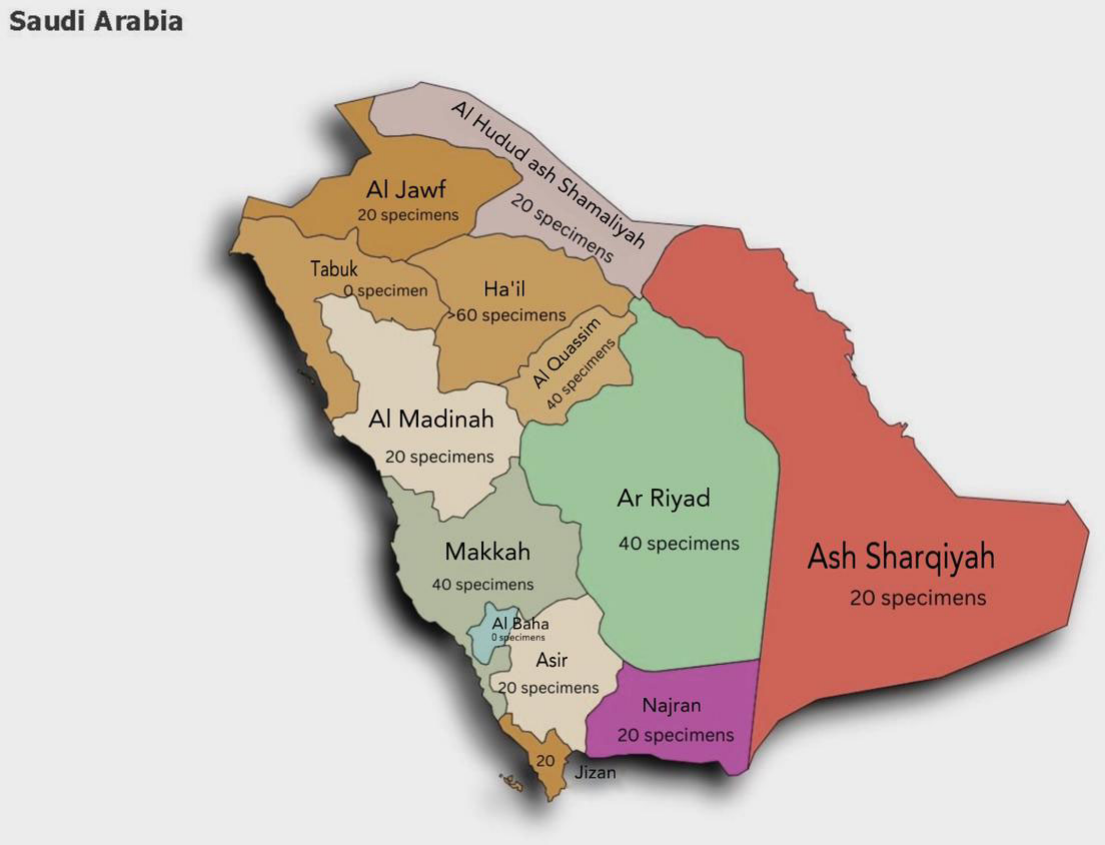
Fungal demand based on data presented from all Saudi regions.

Among Saudi regions, the average number of fungal specimens received per month was 26 samples (**Figure 1**). Most fungal samples were sent from intensive care units (n=7, 30%) followed by dermatology departments (n= 6, 26%), obstetrics and gynecology departments (n=5, 22%), and long-term care units (n=2, 9%). The most common fungal pathogens were *Candida* spp. (n=15, 65%), *Aspergillus* spp. (n= 4, 17%), *Dermatophyte* spp. (n=2, (9%), *Trichophyton* spp. (n=1, 4%), and *Cryptococcus* spp. (n=1, 4%). All hospitals indicated that skin and nail scrapings were the most sent samples for fungal investigations (n=11, 35%), followed by swabs (n=8, 26%), urine (4, 13%), and sputum (n=3, 10%), while vaginal, biopsy, wound, hair, and blood were 3% each (n=1) (**Table 2**). Fungal investigation for BAL samples was not common, with seven hospitals (39%) indicating they received less than five samples per month in the last 12 months, while ten hospitals (56%) did not receive any in the past 12 months. Only one hospital (6%) received 25-50 per months in this period. Biopsy Fresh frozen tissue specimen (BFFT) and Formalin-Fixed Paraffin-Embedded (FFPE) were rare in most hospitals as 89% and 83% of them received less than five samples per month in the last 12 months. Most hospitals did not reject samples (n=12, 67%) in this period. Five hospitals (28%) reported rejecting 1-50 samples per month in this period due to improper storage (n=4, 17%) and improper collection (n=3, 13%). Other rejection reasons were incorrect labeling, incorrect sample size or sample type, incorrect order, or delayed samples (n=1, 4%).

**Table 2:** Survey responses from microbiology laboratories in KSA.

	Number response (n) percentage (%)
Fungal load	
Most common fungal pathogen	
*Candida* spp.	15 (65%)
*Aspergillus* spp.	4 (17%)
*Dermatophyte* spp.	2 (9%)
*Trichophyton* spp.	1 (4%)
*Cryptococcus* spp.	1 (4%)
Number of fungal specimens received/month	
0-20	11(61%)
21-40	5 (28%)
41-60	1(6%)
> 60	1(6%)
Hospital department with highest number of samples	
Intensive care unit	7 (30%)
Dermatology	6 (26%)
Obstetrics and gynecology	5 (22%)
Long-term care	2 (9%)
Transplantation and hematology	1 (4%)
Urology	1 (4%)
Surgical ward	1 (4%)
Most common sample type	
Skin and nail scraping	11 (35%)
Swabs	8 (26%)
Urine	4 (13%)
Sputum	3 (10%)
Vaginal	1 (3%)
Biopsy	1 (3%)
Wound as pus	1 (3%)
Hair	1 (3%)
Blood	1 (3%)
Lowest received sample for fungal investigation	
CSF	8 (29%)
Urine	5 (18%)
Biopsy	5 (18%)
Swabs	2 (7%)
Blood	2 (7%)
Urine from catheterized patient	1 (4%)
Skin or nail scraping	1 (4%)
Sputum	1 (4%)
All mycological samples	3 (11%)
Number of BAL specimens received in the past 12 months	
none	10 (56%)
<5/month	7 (39%)
25-50/month	1 (6%)
Biopsy Fresh frozen tissue specimen received in the past 12 months	
<5/month	16 (89%)
5-25/month	1 (6%)
none	1 (6%)
FFPE specimen received in the past 12 months	
none	15 (83%)
5-25/month	1 (6%)
<5/month	2 (12%)
Laboratory Personal performing fungal detection	
Specialty	
Microbiologist	15 (83%)
General laboratory specialist	3 (17%)
Years of experience	
>10	12 (67%)
<10	6 (33%)
Certificates	
Bachelor’s	6 (33%)
Master’s	6 (33%)
PhD	3 (17%)
Saudi board	2 (11%)
Diploma	1 (6%)
Requesting external consultation	
Yes, I would ask if there was any discrepancy.	11 (61%)
No, I would rather not ask.	6 (39%)
Presence of at least one fungal expert personnel	
Yes	15 (83%)
No	3 (17%)
Maximum mold identification	
Genus	8 (44%)
Genus/species	5 (28%)
N/A	4 (22%)
Can identify 40% by complex	1 (6%)
Maximum yeast identification	
Genus	8 (44%)
Genus/species	8 (44%)
N/A	1 (6%)
Up to 70% of the samples identified by complex	1 (6%)
Laboratory equipment and reagents	
Biosafety cabinet (CLASS II)	
Yes	17 (94%)
No	1 (6%)
Separate incubator for fungal culture	
No	12 (67%)
Yes	6 (33%)
Available direct microscopy and staining method	
Gram stain	15 (31%)
KOH preparation	14 (29%)
Lactophenol cotton blue stain	9 (19%)
India ink stain	4 (8%)
Grocott’s methenamine silver stain	3 (6%)
Fluorescent	2 (4%)
Silver stain	1 (2%)
ITS or 18s identification (molecular/sequencing)	
No	18 (100%)
Available mycological tests	
Semi-automated identification kit	11 (33%)
Manual biochemical methods	6 (18%)
Chromogenic medium	4 (12%)
None	2 (6%)
PCR (commercial for limited species)	2 (6%)
Automated identification system	3 (9%)
Cryptococcus Ag	1 (3%)
Germ tube—manual	2 (6%)
Galactomannan Ag	1 (3%)
MALDI-TOF	1 (3%)
Media for fungal culture	
Sabouraud dextrose agar	18 (60%)
Blood agar	3 (10%)
Sabouraud + chloramphenicol	2 (7%)
CHROM agar	2 (7%)
Mycocel agar	2 (7%)
Dermatophytes media (DTM)	1 (3%)
Sabouraud + gentamicin	1 (3%)
Potato dextrose	1 (3%)
Routine used tests for mycological investigation	
Fungal culture	15 (50%)
Microscopic examination only	15 (50%)
Outsourced tests	
Antifungal susceptibility testing	4 (16%)
PCR	3 (12%)
Culture	4 (16%)
*Cryptococcus* antigen	1 (4%)
MALDI-TOF	1 (4%)
All mycology samples	1 (4%)
BDG	1 (4%)
Fungal biomarkers	1 (4%)
GM Ag	1 (4%)
None	8 (32%)
Performing AST	
No, we did not perform AST	7 (39%)
Yes, we performed AST for yeast only	7 (39%)
Yes, we performed AST for yeast and mold	4 (22%)
AST Method	
Automated MIC for yeast tests	12 (50%)
Disk diffusion method	2 (8%)
E-test	1 (4%)
None	9 (38%)
Diagnostic criteria and quality	
Identified turnaround time as priority	
2 - 9 (50%)	
3 - 6 (33%)	
1 - 3 (17%)	
Identified susceptibility as (primary, secondary, tertiary) decision factor	
1 - 14(78%)	
2 - 2 (11%)	
3 - 2 (11%)	
Identified cost as (primary, secondary, tertiary) decision factor	
3	10 (56%)
2	7 (39%)
1	(6%)
The ability to detect to the species level	
Strong need	10 (56%)
Needed	3 (17%)
Limited need	2 (11%)
Not a strong need	1 (6%)
Would add a comprehensive fungal diagnostic test for	
1-5 samples/month	1 (34%)
> 10 samples/month	4 (22%)
Would not order	8 (44%)
Rejected samples in the last 12 months	
(1-50)	5(28%)
(200-250)	1(6%)
None	12(67%)
Reason of rejection	
Improper storage	4 (17%)
Improper collection	3 (13%)
Unlabeled	1 (4%)
Incorrect sample size or incorrect sample type	1 (4%)
Incorrect order or delayed samples	1 (4%)
None	13 (57%)
Use of quality controls and control strains	
Yes	15 (83%)
No	3 (17%)
Type of accreditation	
CBAHI*	15 (60%)
JCI**	5 (20%)
CAP***	2 (8%)
None	3 (12%)
Perform screening tests for asymptomatic patients	
No	11 (61%)
Yes	7 (39%)
Awareness of fungal infections as serious threat	
More than malaria or breast cancer	13 (72%)
Less than malaria or breast cancer	5 (28%)
Current tools for improvement of knowledge and technical skills	
By practice	11 (52%)
By reading	6 (29%)
By taking courses	1 (5%)
None	3 (14%)
Involvement in fungal research to advance diagnosis	
No	15 (83%)
Yes	3 (17%)
Interested in fungal research to advance diagnosis	
No	9 (50%)
Yes	9 (50%)
Mycological diagnostic tools adequate for confirming fungal infection	
No	10 (56%)
Yes	8 (44%)
Current space for mycology samples	
Microbiology bench	16 (89%)
Separate bench	1 (6%)
A separate laboratory	1 (6%)
Need to add separate mycology section	
No	9 (50%)
Yes	9 (50%)
Obstacles impairing mycological diagnosis	
Clinicians do not request sample often/no need	13 (54%)
Expensive kits	7 (29%)
Unavailable/insufficient trained staff to handle fungal samples	2 (8%)
Challenges for laboratory technologist to diagnose fungal infections	
Availability of facility	18 (47%)
Reagents and kits	12 (32%)
Good training	8 (21%)

*Saudi Central Board for Accreditation of Healthcare Institutions (CBAHI).

**Joint Commission International (JCI).

***College of American Pathologists (CAP).

### 3.3 Laboratory personal performing mycological diagnosis

Most technologists were aware of the global fungal threat and recognized that fungal infections were more common than malaria and breast cancer (n=13, 72%) (**Table 2**). Most of the survey participants were microbiologists (n=15, 83%) with more than 10 years’ experience (n=12, 67%). Most surveyed staff had both bachelor’s and master’s degrees (n=6, 33%). Three had PhD degrees (17%), two had Saudi board (11%), and one had diploma (6%). Most hospitals (n=15, 83%) had at least one expert mycologist who could identify the mold-to-genus level (n=8, 44%) and the species level (n=5, 28%), and one could identify 40% by complex (n=1, 6%). For yeast, most mycologists could offer maximum identification up to the genus (8, 44%) and species (8, 44%) levels and up to 70% of the yeast identified by complex (n=1, 6%) (**Table 2**). For complicated cases, Saudi mycologist always request external consultation (n=11, 61%). They work on improving their technical skills and knowledge by practice (n=11, 52%), reading (n=6, 29%), and attending courses (n=1, 5%). The main challenges for laboratory technologists to diagnose fungal infections are the availability of facilities (n=18, 47%), reagents and kits (n=12, 32%), and good training (n=8, 21%). Three percent of the surveyed mycologists were involved in fungal research, and 50% (n= 9) were interested in research that would advance fungal diagnosis.

### 3.4 Laboratory space and fungal diagnostic services

Many of the hospitals indicated a lack or insufficiency of mycological diagnostic tools in their laboratories (n=10, 56%). Most fungal diagnoses were processed and analyzed on a microbiology bench (n=16, 89%) rather than a separate bench (n=1, 6%) or dedicated laboratory (n=1, 6%) (**Table 2**). Almost all hospitals indicated use of a biosafety cabinet (CLASS II) (n=17, 94%). All hospitals routinely used both microscopic examination and culture tests for mycological investigation 15 (50%). The majority used a plan comprising Sabouraud dextrose agar (n=18, 60%), then blood agar (n=3, 10%), Sabouraud with chloramphenicol (n=2, 7%), CHROM agar (n=2, 7%), and Mycocel agar (n=2, 7%). Rarely, hospitals used dermatophytes media (DTM) (n=1, 3%), Sabouraud with gentamicin (n=1, 3%), and potato dextrose (n=1, 3%). Fungal cultures were incubated at 37°C with a bacterial incubator in 67% of the surveyed hospitals (n=12), and only 33% (n=6) incubate them separately at 20-30°C. Fifty percent of mycologists indicated their need for a dedicated section for mycological investigation (n=9, 50%). Of the hospitals that processed fungus samples, 47% (n=7) reported a turnaround time (TAT) of 2 to 4 weeks, 40% (n=6) reported1 week, and 13% (n=2) reported 4 weeks. For microscopical examination, the following staining techniques were used: Gram stain (n=15, 31%), potassium hydroxide (KOH) preparation (n=14, 29%), lactophenol cotton blue stain (LPCB) (n=9, 19%), India ink stain (n=4, 8%), Grocott’s methenamine silver stain (GMS) (n=3, 6%), calcofluor white stain (n=2, 4%), and silver stain (n=1, 2%).

Other identification tests were included by request, such as a semi-automated identification kit (n=11, 33%), matrix-assisted laser desorption/ionization–time-of-flight (MALDI-TOF) (n=1, 3%), automated identification system (n=3, 9%), manual biochemical methods (n=6, 18%), germ tube test—manual (n=2, 6%), chromogenic medium (n=4, 12%), PCR (commercial for limited species) (n=2, 6%), galactomannan (GM)Ag (n=1, 3%), and *Cryptococcus* Ag (n=1, 3%), while other hospitals did not perform any of the previously mentioned tests (n=2, 6%) (**Table 2**). None of the hospitals performed sequencing for fungal identification, such as nuclear ribosomal internal transcribed spacer (ITS) or 18S rRNA gene (n=18, 100%). Many tests were outsourced to private laboratories, including antifungal susceptibility testing (AST) (n=4, 16%), PCR (n=3, 12%), culture (n=4, 16%), *Cryptococcus* antigen (Bongomin et al., 2017; Wickes and Romanelli, 2020),-β-d-glucan (BDG), GM (n=1, 4%), MALDI-TOF (n=1, 4%), and all mycology samples (n=1, 4%), while others did not (n=9, 36%). AST was performed abundantly for yeast only (n=7, 39%), though few hospitals had AST for both mold and yeast (n=4, 22%), and many did not perform the test (n=7, 39%). The most used AST method was automated minimal inhibitory concentration (MIC) for yeast tests (n=12, 50%), followed by the disk diffusion method (n=2, 8%) and E-test (n=1, 4%), though many did not perform the test (n=9, 38%). Most of mycological diagnostic laboratories used quality controls and control strains (n=15, 83%), while only three (17%) did not. Screening for high-risk asymptomatic patients was not performed in many hospitals (n=11, 61%), though a few did (n=7, 39%).

### 3.5 General challenges and improvements

To improve fungal detection, we examined three factors: susceptibility, turnaround, and cost. Most mycologists prioritized the identified susceptibility as primary (n=14, 78%), while others thought it was secondary or tertiary (n= 2, 11%). The turnaround time was the secondary priority (n=9, 50%), while cost played a tertiary factor for adding new assays (n=10, 56%) (**Table 2**). Other obstacles impairing mycological diagnosis included empirical treatment without sending a sample for fungal investigation (n=13, 54%), expensive kits/unable to provide (n=7, 29%), and unavailable/insufficient trained staff to handle fungal samples (n=2, 8%). Based on the current fungal load, most laboratories would not need a comprehensive fungal assay (n=8, 44%). Other hospitals would order more than 10 samples per month (n=4, 22%) or 1 to 5 samples per month (n=1, 34%) (**Table 2**). A large percentage of hospitals had a strong need to provide assays that provide genus/species identification (n=10, 56%), while fewer needed (n=3, 17%) or had a limited need (n=2, 11%), and one hospital did not have a need (n=1, 6).

## 4 Discussion

This study intended to investigate the gap in fungal diagnosis and compare challenges reported in the literature with those in national Saudi hospitals. The distribution of fungal infections between geographic areas is affected by environmental conditions, climate, travel, and population. Although Saudi Arabia is known for its desert environment, which is extremely hot and dry in the summer and cold and dry in the winter, the southwest has a semi-arid climate, and the west and east coasts have increased humidity. Saudi Arabia has a hot and humid climate in the summer. As a result, dermatophytoses such as *tinea corporis* and *tinea cruris* were discovered to be the most frequent types of dermatophytosis in the Eastern Province of Saudi Arabia (Al-Sogair et al., 1991). The likelihood of dermatophytes infections is present in almost all seasons, as reported in another Saudi study about an increase of dermatophytosis seasonal incidence during the winter and spring (Alshehri et al., 2021). These studies explain why nail and skin scrapings are the most common sample types that underwent fungal investigations in our study. All the participating hospitals rely on microscopic examination to diagnose this type of fungal infections, as shown in the results. The microscopic examination of dermatophytes has 90.5% accuracy (Aboul-Ella et al., 2020) Although microscopic examination is the gold standard to diagnose dermatophytes, differentiating dermatophyte hyphae/elements from other fungi requires a high scale of experience (Kidd and Weldhagen, 2022).

The *Candida* genera were the most dominant in all hospitals, agreeing with the global estimates of fungal infections (Bongomin et al., 2017). We anticipate that there is a chance for false negatives or misidentification related to the absence of a dedicated incubator for fungi and the non-standardized incubation temperature represented by some of the participated laboratories. Molds are more challenging to diagnose compared to yeasts. Generally, fungal culture requires an extended incubation time for growth compared to yeasts. Furthermore, culture difficulties were reported in 17 cases, with 7 cultures positive with IA from hematologic patients, especially the culture of *Aspergillus* from respiratory specimens, which may have been missed (Althoff Souza et al., 2006). Serological tests such as GM were not widely accessible for diagnosing *Aspergillus* infections, and cross-reactivity has been observed in immunocompromised patients (Barton, 2013). Similarly, assessing mold diagnosis with molecular tests such as PCR was not commonly used and in some cases outsourced. It is recommended to use molecular advance techniques to improve sensitivity and overcome cross reactivity (Bretagne and Alanio, 2017).

With the increase of the immunosuppressed population in Saudi Arabia, we predict that there will be a rise in the number of fungal infections in this patient population. According to the cancer trends in Saudi Arabia, the most prevalent malignancies were leukemia, colorectal cancer, and Hodgkin’s lymphoma among Saudi men. In Saudi women, the most common were breast, thyroid, and colorectal cancer. Hematological cancers are among the three malignancy categories with the highest prevalence. However, the present gaps lead to an increase in false negative results, thus causing an underestimate of fungal infection reports. For example, basidiobolomycosis is a known as a rare fungal infection that affects immunocompetent young adults and rarely affects the gastrointestinal tract. In the past few years, many Saudi Arabian studies have reported cases of *Basidiobolus ranarum*, with most reported cases in children and the majority coming from the southern region of Saudi Arabia (Alshehri, 2013). None of the southern hospitals reported detecting *Basidiobolus ranarum.* The gold standard diagnostic test for distinguishing these mycotic lesions from malignancy is using histopathological methods and species confirmation by culture or polymerase chain reaction (Vs et al., 2021). Biopsies must be sent to the microbiology laboratory to complete the required diagnosis (Bretagne and Alanio, 2017). According to our results, only few biopsies are sent to the microbiology department for fungal investigation. However, in many cases, the tissue samples were sent in formalin, killing all microorganisms. Sixteen percent of the participants indicated good training was a challenge, and five percent reported a lack of knowledge. The responsibility of sending the required samples falls upon the healthcare team and policymakers. The diagnosis of fungal infection requires a wide index of suspicion, good training, and greater understanding of some rare condition aids in early detection. In mycology laboratories, this can be achieved by following a standardized diagnostic scheme for early diagnosis and assessing the validity for diagnostic results to implement a precise medical intervention and to avoid delay of the treatment.

We asked the participants to evaluate three factors, namely, susceptibility testing, turnaround time, and cost, based on their diagnostic need. Most participants chose susceptibility testing as the first choice, which shows a good adherence to mycology practice. Susceptibility testing became important in healthcare settings to track the transmission of some well-known multi-drug-resistant organisms such as *Candida auris*. Outbreaks of *C. auris* have been reported globally and in Saudi Arabia. A further challenge is that proper identification of *C. auris* needs specialized laboratory methods. Misidentification and incorrect treatment of this fungus are common in healthcare facilities due to obsolete or outdated techniques, rendering it difficult to control its spread. Local healthcare authorities and laboratories need to ensure that proper diagnostic methods are used to detect *C. auris* to reduce its transmission circle, as suggested by the CDC (The Centers for Disease Control and Prevention (CDC), 2022). According to our results, most local laboratories depend on semi-automated identification kits to perform fungal susceptibility testing. More efforts should be directed to prevention as healthcare systems shift toward routine screening for *C. auris* colonization (Kordalewska and Perlin, 2019). While MALDI-TOF can correctly identify *C. auris* (Shallu K et al., 2015), biochemical reaction-based commercial methods such as Vitek 2 and API tests can misidentify *C. auris* (Ding et al., 2019). Misidentification is one of the obstacles that delays treatment interventions and causes long TAT for the growth of fungal culture. The association between hospital mortality and the time of antifungal treatment after the reporting of a positive culture for candidemia shows higher mortality risks after more than 12 hours’ delay of treatment (Morrell et al., 2005). Therefore, the need for cost-effective diagnostic tools that provide a rapid result is crucial. According to our results, most laboratories lack fungal selective culture media, fungal serological tests, and lateral flow, which were not available in all the laboratories as screening or monitoring tests. Although cost is a dominant factor from the direct purchasers’ perspective, it is important to highlight the national precautions being undertaken against the growing incidence of fungal infections when considering allocating an adequate budget for fungal tests.

Some questions in our survey were designed to investigate the opinions of laboratory specialists about the fungal diagnostic status in their institutions. The responses emphasized the need for fungal diagnostic improvements, with half of the participants suggesting a dedicated place for fungal samples and advanced diagnostic tests such as next-generation sequence (NGS), which provide prompt results compared to conventional methods. We closed the survey by asking the participants about the challenges they most encounter as laboratory specialists in diagnosing fungal infections, and most of the answers ranged from lack of equipment and facilities or diagnostic tests to insufficient knowledge and limited mycology experts. We believe that fungal diagnostics can be improved if the challenges and required diagnostic needs are addressed.

This is the first cross-sectional study in Saudi Arabia investigating the current status of fungal diagnosis and the general need for local mycological diagnosis in Saudi hospitals. Although 280 hospitals were contacted, only 57 participants were able to complete part of the survey. This was due to lack of facilities or staff or because fungal detection was not performed. There was also a limitation in using call interviews as participants’ answers were approximate, which may point to recall bias. The hypothetical and opinion questions were prone to self-report and social desirability biases as some participants may have felt pressure. According to the population load, there was a good representation of all regions of Saudi Arabia. Diagnosing fungal infections is still difficult due to the distinctive nature of fungi and the relative frequency of infection compared to bacteria. This has created considerable challenges for developing rapid, comprehensive assays for fungal identification and implementing them globally (Wickes and Romanelli, 2020). Based on this study, the following recommendations are advised for better fungal detection: (i) to establish an active fungal reference laboratory equipped with all the required tests; (ii) to acquire rapid diagnostic tests with long shelf lives; (iii) to reconsider fungal diagnostic schemes and external quality assessment programs specialized for fungi; (iv) to increase awareness of fungal diseases by offering fungal courses, seminars, and workshops to healthcare teams; (v) to adapt more fungal research to understand and implement novel technologies to solve the current clinical fungal diagnostic problems; and (vi) to dedicate national open-source documentation in all medical institutions for fungal infections.

## Data availability statement

The original contributions presented in the study are included in the article/**Supplementary Material**. Further inquiries can be directed to the corresponding authors.

## Ethics statement

This project was approved by the Ethical Committee of Scientific Research at the College of Applied Medical Sciences, Taibah University. Consent signatures were collected electronically in the survey invitation forms.

## Author contributions

AMK was responsible for the organization, design, and coordination of the study and was also the chief investigator and responsible for the data analysis. SA conducted the interviews and oversaw the initial data analysis. All authors contributed to the writing of the final manuscript.
