# Supplementary material for: Cross-sectional investigation of mycological diagnosis challenges in Saudi Arabia
Source: Front Cell Infect Microbiol. 2023 Jun 12;13:1203892. doi: 10.3389/fcimb.2023.1203892 (PMC10332264; doi:10.3389/fcimb.2023.1203892)
Supplement: Supplementary file 1 [file Table_1.docx]

**Cross-sectional Investigation of Mycological Diagnosis Challenges in Saudi Arabia**

**Supplementary materials**

**Table S1.** Survey questions of mycological diagnosis challenges in Saudi Arabia

| **Time:**  **Phone\respondent no. :**  **Hospital #:**  **E-mail:                                                                 filled by:**  ***DEMOGRAPHY***   1. **What is your specialty? (multiple choice)**  - Microbiology - Histopathology  1. **Where is your job location? (multiple choice)**  - Riyadh region - Mecca region - Medina region - Al-Qassim region - Eastern region - Asir region - Tabuk region - Northern borders region - Jizan region - Najran region - Al-Baha region - Al-Jouf region - Hail region  1. **Which of the following is your affiliated institution type? (multiple choice)**  - University hospital - Public Hospital - Private Hospital - Health Center - Clinic - Research Hospital - Others (please specify)__________________________   ***SAMPLE EVALUATION***   1. **How many fungal specimens do you often receive in your laboratory per month? (Multiple choice)**  - 1-10 - 11-20 - 21-30 - Others (please specify)__________________________  1. **During the past 12 months, the majority of samples were sent from (checkbox)**  - Intensive care unit (ICU) - Surgical ward - Obstetrics & Gynecology (OG) - Outpatient department (OPD) - Others (please specify)__________________________  1. **What are the *most* fungal samples did you receive in the past 12 months?(checkbox)**  - Skin or nail scraping - Attached devices (i.e catheter, bandages…) - Swabs - Blood - Urine - Sputum - BAL - Biopsy: Fresh tissue - Biopsy: frozen tissue - CSF   Others (please specify)__________________________   1. **What are the *least* fungal samples did you receive in the past 12 months (check box)**  - Skin or nail scraping - Attached devices (i.e catheter, bandages…) - Swabs - Blood - Urine - Sputum - BAL - Biopsy: Fresh tissue - Biopsy: frozen tissue - CSF - Others (please specify)__________________________   ***LABORATORY SPACE & EQUIPMENT***   1. **Does your laboratory participate in any fungal External Quality Assessment Schemes (EQAS)? (check box)**  - Yes, we have (provide the accreditation name please). - MOH - CBAHI - CAP - Others (please specify) _______________________________ - No, we do not.      1. **Do you have a mycology section dedicated for mycology samples or do you process it in a microbiology bench? (check box)**  - Yes, we have a separate bench. - No, we process in the microbiology bench. - Others (please specify)__________________________     *9-1. (NO*) **Do you think you need to add a separate mycology section? (check box)**   - No, I do not need to. - Yes, I do. **(what is stopping you?)** - Clinicians do not request samples often. - Reagents not available. - Expensive kits. - No dedicated incubator. - Unavailable\ insufficient trained staff to handle fungal samples. - Others (please specify)__________________________      1. **Do you have a biosafety cabinet (CLASS II) for fungal diagnostic tests? (multiple choice)**  - Yes, we have a (CLASS II) biosafety cabinet. - No, we do not have a (CLASS II) biosafety cabinet.  1. **Do you have a separate incubator for fungal diagnostic tests? (multiple choice)**  - Yes, we have. (what is the temperature?)____________________ - No, we do not.   ***FUNGAL DIAGNOSTIC TESTS***   1. **During the past 12 months, what is the main test you perform for the mycological specimens? (check box)**  - Fungal culture - Microscopic examination - Germ tube test - Molecular tests - PCR - Next Generation Sequencing (NGS) - Serological tests **(if yes please choose from the following)**   o   Cryptococcal antigen detection assays  o   *Candida* antigen detection assays  o   *Candida* antibody detection assays  o   Galactomannan assays  o   *Aspergillus* antibody detection assays  o   Beta-D-Glucan (BDG)   - Others (please specify)__________________________      1. **Mycological tests available in your laboratory during the past 12 months (check box)**  \|  \| Chromogenic medium \| \| --- \| --- \| \|  \| Manual biochemical methods \| \|  \| Semi-automated identification kit \| \|  \| Automated identification system \| \|  \| Lateral flow \| \|  \| MALDI-TOF \| \|  \| BDG AgG \| \|  \| Cryptococcus antigen \| \|  \| GM Ag \| \|  \| DNA sequencing \| \|  \| Next Generation Sequencing (NGS) \| \|  \| PCR \| \|  \| Others (please specify) _______________________________ \|      1. **Which methods do you use during the past 12 months for fungal cultures (check box)**  \|  \| Sabouraud agar \| \| --- \| --- \| \|  \| Sabouraud + Chloramphenicol \| \|  \| Sabouraud+ Gentamicin \| \|  \| Dermatophytes media (DTM) \| \|  \| Potato dextrose \| \|  \| Corn meal (corny) media \| \|  \| Others (please specify)___________________________ \|      1. **Which of the following staining methods were available in your laboratory in the past 12 months: (check box)**  \|  \| KOH preparation \| \| --- \| --- \| \|  \| India ink stain \| \|  \| Lactophenol cotton blue stain \| \|  \| Gram stain \| \|  \| Grocott's methenamine silver stain \| \|  \| Fluorescent stain \| \|  \| Others (please specify) _______________________________ \| \|  \| None \|      1. **During the past 12 months, did you perform Antimicrobial Susceptibility Testing (AST)? (multiple choice)**  - Yes, we performed AST for yeast only. - Yes, we performed AST for mold only. - Yes, we performed AST for  yeast and mold. - No, we did not perform AST. - Others (please specify) _______________________________      1. **Which of the following Antimicrobial Susceptibility Tests (AST) were available in your laboratory during the past 12 months (check box)**  \|  \| Disk diffusion method \| \| --- \| --- \| \|  \| MIC \| \|  \| Sensititre YeastOne \| \|  \| E-test \| \|  \| VITEK tests \| \|  \| Beta-2-glycoprotein test (B2G) \| \|  \| Others (please specify) ___________________________ \|      1. **During the past 12 months, which fungal tests you need but outsourced to another hospital or private diagnostic laboratories (check box)**  \|  \| AST \| \| --- \| --- \| \|  \| MALDI-TOF \| \|  \| DNA sequencing \| \|  \| Serology testing (Cryptococcus antigen, GM Ag,BDG AgG) \| \|  \| Others (please specify)____________________________________ \| \|  \| None, we do not refer any samples to another hospital for identification \|      1. **During the past 12 months, what are the most common pathogens in your hospital (check box)**  - *Aspergillus spp.* - *Candida spp.* - *Cryptococcus spp.* - *Basidiomycota* - *Fusarium spp.* - *Histoplasma spp.* - *Mucorales* - Others (please specify)___________________________      1. **What is the maximum identification capability of molds in your laboratory? (multiple choice)**  - Genus - Genus \species - Genus \species \ complex - Genus \species \ complex \ cryptic species  1. **What is the maximum identification capability of yeast in your laboratory? (multiple choice)**  - Genus - Genus \species - Genus \species \ complex - Genus \species \ complex \ cryptic species      1. **How many years of experience do you have? (multiple choice)**  - 2 - 4 - 6 - Others (please specify)__________________________      1. **Did you obtain any of the certificates below? (multiple choice)**  - Saudi board - American board - ASCP License - Others (please specify)__________________________      1. **How do you improve your knowledge and technical skills in fungal diagnosis?**  - By practice - By taking courses, (name of the course_______________________) - None - Others (please specify)__________________________      1. **Based on the last 12 months, what is your realistic turnaround time (TAT) expectations/needs for your fungal pathogen assays?  (NOTE: TAT = "time from collection to receipt of results”) (multiple choice)**      - 1-2 days - 3-4 days - 5-7 days - 8-10 days - Others (please specify)___________________________      1. **Based on the last 12 months, how strong is your need for an assay that provides genus and species fungal results? (multiple choice)**  - Not a strong need. - Limited need. - Needed. - Strong need. - Please elaborate__________ - Others (please specify)___________________________      1. **How often would you consider ordering a comprehensive fungal diagnostic test, like Next Generation Sequencing (NGS), if it provided genus species results within 73 hours of collection? (multiple choice)**  - Would not order - <1 / month - 1-5 / month - 6-10 / month - > 10 / month - Others (please specify)___________________________      1. **Based on your experience,  please rank by importance the diagnostic goal in fungal infections. (use number 1-3 , 1 being most important) (rank)**  - **TAT**  \| **1** \| **2** \| **3** \| \| --- \| --- \| --- \|  - **Susceptibility**  \| **1** \| **2** \| **3** \| \| --- \| --- \| --- \|  - **Cost**  \| **1** \| **2** \| **3** \| \| --- \| --- \| --- \|  1. **Do you think the mycological diagnosis in your hospital is adequate for conforming fungal infection? (multiple choice)**  - Yes, it is enough - No, it is not enough **(what do you suggest to improve the mycological diagnosis in your hospital?)_________________** - Others (please specify)___________________________  1. **In your opinion, what challenges do laboratory technologists encounter to diagnose fungal infections?**   **_________________________**   1. **Do you think fungal infections are………..malaria and breast cancer?**  - More than - less than  1. **Do you have any other comments or ideas about fungal diagnostics you would like to share?**   **____________________________** |
| --- | --- | --- | --- | --- | --- | --- | --- | --- | --- | --- | --- | --- | --- | --- | --- | --- | --- | --- | --- | --- | --- | --- | --- | --- | --- | --- | --- | --- | --- | --- | --- | --- | --- | --- | --- | --- | --- | --- | --- | --- | --- | --- | --- | --- | --- | --- | --- | --- | --- | --- | --- | --- | --- | --- | --- | --- | --- | --- | --- | --- | --- | --- | --- | --- | --- | --- | --- | --- | --- | --- | --- | --- | --- | --- | --- | --- | --- | --- | --- | --- | --- | --- | --- | --- | --- | --- | --- | --- | --- | --- | --- |
